# Predicting multiple mental health outcomes in adolescents using explainable machine learning models

**DOI:** 10.3389/fpsyt.2026.1923390

**Published:** 2026-09-18

**Authors:** Xiaojie Dong, Jing Meng, Peng Wang, Chuandong Yan, Xiaobo Liu

**Affiliations:** 1 Department of Psychiatry, Ward 1, Wuhan Mental Health Center, Wuhan, Hubei, China; 2 Department of Medical Administration, Wuhan Mental Health Center, Wuhan, Hubei, China

**Keywords:** adolescent, emotional dysregulation, loneliness, machine learning, mental health, predictive modeling

## Abstract

**Introduction:**

Adolescent mental health problems, including depression, anxiety, and stress, are an increasing public health concern, yet the factors associated with different mental health outcomes may vary across domains. This study investigated shared and outcome-specific predictive features of depression, anxiety, perceived stress, and psychological well-being using explainable machine learning models.

**Methods:**

A cross-sectional study was conducted among 1,088 adolescents aged 13–18 years recruited from secondary schools in Wuhan, China, using multistage cluster sampling. Participants completed validated measures of emotional dysregulation, loneliness, social media addiction, self-esteem, sleep quality, academic stress, family support, physical activity, and mental health outcomes. Four algorithms—linear regression, support vector regression, Random Forest, and XGBoost—were trained using an 80/20 train-test split with five-fold cross-validation, and model performance was evaluated using test-set R², RMSE, and MAE. SHapley Additive exPlanations (SHAP) were used to examine feature contributions. To minimize target leakage, outcome-specific feature sets were used, with PSQI and RSES excluded from the depression model because of direct or substantial conceptual overlap with PHQ-9 content, and PSQI excluded from the well-being model because of overlap with WHO-5 content.

**Results:**

XGBoost showed the strongest out-of-sample predictive performance across all four outcomes, explaining 49% of the variance in depression (R² = 0.49, 95% CI: 0.44–0.53; RMSE = 3.52; MAE = 2.81), 55% in anxiety (R² = 0.55, 95% CI: 0.50–0.59; RMSE = 3.04; MAE = 2.47), 60% in perceived stress (R² = 0.60, 95% CI: 0.56–0.64; RMSE = 3.71; MAE = 2.87), and 50% in psychological well-being (R² = 0.50, 95% CI: 0.45–0.54; RMSE = 3.38; MAE = 2.68).

**Discussion:**

Emotional dysregulation and loneliness were consistently among the most influential features, while academic stress, family support, social media addiction, self-esteem, and sleep quality showed outcome-specific contributions. SHAP rankings for the depression model were stable across five-fold cross-validation, with emotional dysregulation and loneliness consistently occupying the highest ranks.

## 1 Introduction

Adolescence is a developmental period marked by profound biological, cognitive, and social changes. Although these transitions create opportunities for growth and identity formation, they also increase vulnerability to a range of mental health difficulties (1). Depression, anxiety, and stress-related symptoms are among the most frequently reported psychological concerns during adolescence and have become major public health issues worldwide (2). Mental health problems emerging during this stage often extend into adulthood, affecting educational attainment, interpersonal relationships, occupational functioning, and overall quality of life (3). Consequently, identifying factors associated with adolescent mental health has become an important priority for researchers and practitioners.

Research has consistently shown that different dimensions of mental health are closely interconnected (4, 5). Depression, anxiety, and perceived stress frequently co-occur and share several common psychosocial correlates, whereas positive indicators such as psychological well-being are shaped by both protective and vulnerability-related processes (6). Despite this overlap, many studies continue to examine mental health outcomes separately, making it difficult to determine whether specific psychosocial factors show consistent associations across multiple outcomes or are uniquely associated with particular dimensions of mental health.

Among the factors that have received increasing attention in recent years, emotional dysregulation has emerged as a potentially important transdiagnostic construct. Emotional dysregulation refers to difficulties in understanding, managing, and responding effectively to emotional experiences (7). Adolescents who struggle to regulate their emotions are more likely to experience a variety of psychological difficulties, including depressive symptoms, anxiety, and heightened stress responses (8). Growing evidence suggests that difficulties in emotion regulation represent a transdiagnostic correlate that is consistently associated with diverse forms of psychological distress (9, 10).

Loneliness has also become a prominent concern during adolescence. Although this developmental stage is typically characterized by expanding peer relationships and increased social engagement, many adolescents report persistent feelings of social isolation and unmet relational needs (11, 12). Loneliness has been associated with a wide range of adverse outcomes, including depression, anxiety, lower life satisfaction, and poorer psychological well-being (13, 14). Recent social and technological changes have further intensified interest in loneliness as a psychosocial characteristic associated with a broad range of adverse mental health outcomes among young people (13, 14).

Another factor that has attracted considerable attention is social media addiction. Social media platforms play a central role in adolescents’ daily lives and provide opportunities for communication, self-expression, and social interaction (15). However, excessive and uncontrolled engagement with social media has been linked to emotional difficulties, sleep disturbances, social comparison, and psychological distress (16). Although previous research has documented associations between social media addiction and various indicators of mental health (17, 18), questions remain regarding its relative importance when considered alongside other established psychosocial correlates.

In addition to these factors, several individual, familial, and behavioral characteristics have been repeatedly associated with adolescent mental health. Self-esteem is widely recognized as a protective factor against psychological distress (19), whereas academic stress has been linked to elevated levels of depression, anxiety, and emotional burden (20). Higher levels of perceived family support have been associated with better psychological adjustment and lower psychological distress (21), while physical activity has been associated with improved emotional functioning and mental well-being (22). However, these factors rarely operate independently. Instead, they interact within a complex network of relationships that may influence mental health outcomes simultaneously.

Despite substantial progress in the literature, several important gaps remain. First, much of the existing research has focused on a single mental health outcome, limiting understanding of whether psychosocial adverse and protective psychosocial characteristics operate similarly across different dimensions of mental health. Second, psychosocial characteristics such as emotional dysregulation, loneliness, social media addiction, self-esteem, sleep quality, family support, academic stress, and physical activity are conceptually related and often empirically correlated. Examining these variables in isolation may therefore provide only a partial understanding of adolescent mental health. Third, conventional statistical approaches frequently rely on assumptions of linearity and may be challenged by substantial overlap among predictors, making it difficult to capture complex interactions and nonlinear relationships.

Machine learning approaches offer a promising framework for addressing these challenges. Unlike traditional regression-based methods, machine learning algorithms are capable of handling large numbers of interrelated predictors and identifying complex patterns that may not be readily detected through conventional analyses (23). This feature may be particularly valuable in adolescent mental health research, where psychosocial characteristics are often highly interconnected. At the same time, concerns regarding the interpretability of machine learning models have led to growing interest in explainable artificial intelligence techniques. Methods such as SHapley Additive exPlanations (SHAP) allows researchers to examine the relative importance of model features and better understand how individual variables contribute to model predictions (24).

Although machine learning has been increasingly applied in mental health research, relatively few studies have simultaneously examined multiple mental health outcomes while investigating the relative importance of psychosocial characteristics. Furthermore, the comparative roles of emotional dysregulation, loneliness, and social media addiction within a unified predictive framework remain insufficiently understood.

To address these gaps, the present study integrates multiple machine learning algorithms with explainable artificial intelligence to simultaneously model multiple adolescent mental health outcomes within a unified analytical framework. Most prior studies have tended to focus on single mental health outcomes, which limits understanding of how psychosocial factors operate across different domains of adolescent mental health. By incorporating SHapley Additive exPlanations (SHAP), the study further enhances interpretability, allowing for a transparent understanding of how complex behavioral and psychological factors are jointly associated with adolescent mental health profiles.

Therefore, the present study aimed to develop and compare explainable machine learning models for modeling variation in depression, anxiety, perceived stress, and psychological well-being among adolescents. Particular attention was given to the relative importance of emotional dysregulation, loneliness, social media addiction, self-esteem, sleep quality, academic stress, family support, and physical activity as candidate predictive features. By comparing feature importance across multiple mental health outcomes, the study sought to identify both shared and outcome-specific psychosocial characteristics associated with adolescent mental health.

## 2 Methods

### 2.1 Study design and participants

A cross-sectional study was conducted among adolescents enrolled in secondary schools in Wuhan, China. Participants were recruited using a multistage cluster sampling approach. In the first stage, districts were stratified into urban and suburban regions. In the second stage, six secondary schools (three junior high schools and three senior high schools) were randomly selected from the official list of eligible public schools provided by the Wuhan Municipal Education Bureau. Schools were sampled to ensure representation from both urban and suburban districts. The selected schools represented a range of academic achievement levels according to local educational classifications, thereby improving the diversity of the study sample. In the third stage, classes within each selected school were chosen using a cluster sampling procedure. Finally, all students from the selected classes were invited to participate.

Data collection was conducted during regular school hours between August and November 2025. Research assistants received standardized training before data collection to ensure consistent questionnaire administration across all participating schools. Trained research assistants administered the questionnaires in classroom settings using standardized instructions. Completion of the survey package required approximately 25–30 minutes. Questionnaires were reviewed immediately after collection to minimize missing responses. Data were subsequently entered into a secure database and checked for accuracy before analysis.

Students were eligible if they were between 13 and 18 years of age, enrolled in one of the participating schools, and able to complete the questionnaires independently. Students with diagnosed cognitive impairments that prevented questionnaire completion and those with substantial missing responses (> 20% of survey items) were excluded.

Sample size was estimated using G*Power version 3.1. Based on a multiple regression model with eight primary predictors, a small-to-moderate effect size (f² = 0.04), an alpha level of 0.05, and a statistical power of 0.95, the minimum required sample size was calculated to be 787 participants. Although this baseline estimate relied on traditional multiple regression assumptions, the study’s objective of developing and comparing multiple machine learning models required a larger sample to prevent excessive variance during cross-validation and ensure stable out-of-sample performance. Therefore, assuming a response rate of approximately 85%, a total sample of approximately 1,200 adolescents was targeted.

Ethical approval for this study was obtained from the Ethics Committee of Tongji Medical College, Huazhong University of Science and Technology, Wuhan, China (No. HUST-25-X218). This work was conducted in accordance with the Declaration of Helsinki. Approval was obtained from school administrators before data collection. Because all participants were younger than 18 years of age, written informed consent was obtained from parents or legal guardians. In addition, participating students provided written assent after receiving detailed information regarding the study objectives, confidentiality of responses, voluntary participation, and their right to withdraw at any stage without academic consequences. No personally identifiable information was collected, and all responses were analyzed anonymously.

### 2.2 Measures

Internal consistency was evaluated using Cronbach’s alpha. In the present sample, coefficients ranged from 0.67 to 0.81, indicating acceptable reliability for most instruments, although the perceived family support (MSPSS-Family; α = 0.67) and physical activity (PARS-3; α = 0.68) subscales demonstrated internal consistency slightly below the conventional 0.70 threshold. Because both constructs have been consistently associated with adolescent mental health in previous research, they were retained in the analyses. However, the predictive contributions of these variables should be interpreted cautiously because lower internal consistency may have reduced the reliability of these measurements and potentially attenuated their predictive associations.

### 2.3 Mental health outcomes

Depressive symptoms were assessed using the Chinese version of the Patient Health Questionnaire-9 (PHQ-9) (25). The PHQ-9 consists of nine items rated from 0 (not at all) to 3 (nearly every day), with total scores ranging from 0 to 27. Higher scores indicate greater depressive symptom severity. The Chinese version has demonstrated good psychometric properties among adolescents. Anxiety symptoms were measured using the Chinese version of the Generalized Anxiety Disorder Scale-7 (GAD-7) (26). The scale contains seven items scored from 0 to 3, yielding total scores between 0 and 21. Higher scores reflect greater anxiety symptoms. Perceived stress was assessed using the Chinese version of the Perceived Stress Scale-10 (PSS-10) (27). The instrument includes ten items rated on a five-point Likert scale ranging from 0 (never) to 4 (very often). Higher scores indicate greater perceived stress. Psychological well-being was measured using the Chinese version of the WHO-5 Well-Being Index (28). The WHO-5 contains five items scored from 0 to 5, with higher scores indicating better psychological well-being.

#### 2.3.1 Psychosocial predictors

Emotional dysregulation was assessed using the Chinese version of the Difficulties in Emotion Regulation Scale–Short Form (DERS-SF) (29). The scale comprises 18 items rated on a five-point Likert scale, with higher scores indicating greater difficulties in emotion regulation. Loneliness was measured using the Chinese version of the UCLA Loneliness Scale (Version 3) (30). The instrument consists of 20 items scored on a four-point Likert scale. Higher scores represent greater perceived loneliness. Social media addiction was assessed using the Chinese version of the Bergen Social Media Addiction Scale (BSMAS) (31). The BSMAS contains six items rated from 1 (very rarely) to 5 (very often), with higher scores indicating greater addictive social media use. Self-esteem was measured using the Chinese version of the Rosenberg Self-Esteem Scale (RSES) (32). The scale contains ten items rated on a four-point Likert scale, with higher scores reflecting greater self-esteem.

#### 2.3.2 Behavioral and contextual factors

Sleep quality was assessed using the Chinese version of the Pittsburgh Sleep Quality Index (PSQI) (33). The PSQI contains 19 self-report items and yields a global score ranging from 0 to 21, with higher scores indicating poorer sleep quality. Academic stress was measured using the Educational Stress Scale for Adolescents (ESSA) (34). The scale includes 16 items rated on a five-point Likert scale, with higher scores indicating greater academic stress. Perceived family support was assessed using the family subscale of the Multidimensional Scale of Perceived Social Support (MSPSS) (35). The subscale consists of four items rated on a seven-point Likert scale. Higher scores indicate stronger perceived family support. Physical activity was measured using the Chinese version of the Physical Activity Rating Scale-3 (PARS-3) (36). The instrument assesses exercise intensity, frequency, and duration through three items and generates a composite score ranging from 0 to 100. Higher scores indicate greater participation in physical activity.

### 2.4 Statistical analysis

Data analyses were performed using SPSS version 29 for descriptive statistics and Python version 3.11 for machine learning modeling. The scikit-learn, xgboost, pandas, numpy, and shap libraries were utilized for model development and interpretation. Descriptive statistics were calculated for all study variables, and Pearson correlation coefficients were used to examine bivariate associations. Continuous variables were inspected for normality using histograms and Q-Q plots, whereas multicollinearity among candidate predictive features was evaluated using variance inflation factors (VIFs) before model development. All VIF values were below 3.0, indicating that problematic multicollinearity was unlikely. Prior to machine learning analyses, data were screened for missing values, outliers, and distributional characteristics. Participants with more than 20% missing responses were excluded.

The dataset was initially randomly partitioned into training (80%) and testing (20%) subsets. To strictly prevent data leakage, all subsequent preprocessing steps were fitted exclusively on the training set and then applied to the test set using the learned transformation parameters. These steps included addressing missing data via Multiple Imputation by Chained Equations (MICE) and standardizing continuous predictor variables using Z-score normalization. Additionally, categorical variables, including gender, were transformed using dummy coding prior to inclusion in the models.

Before model development, the candidate predictors were examined for direct measurement overlap with each outcome measure. Predictors with substantial overlap in item or scale content were excluded from the corresponding outcome-specific model to minimize potential target leakage arising from shared measurement content. For the PHQ-9 model, both PSQI and RSES were excluded. PSQI was excluded because the PHQ-9 includes an item assessing sleep disturbance, which overlaps with sleep-related content assessed by the PSQI. RSES was excluded because an RSES item assessing feelings of being a failure substantially overlaps with the PHQ-9 item assessing feelings of worthlessness, failure, or self-blame. For the WHO-5 model, PSQI was excluded because the WHO-5 includes an item assessing waking up feeling fresh and rested, which overlaps with sleep-related content assessed by the PSQI. PSQI remained eligible for the models predicting anxiety and perceived stress because no substantial direct measurement overlap was identified.

Four separate outcome-specific predictive models were developed using depression, anxiety, perceived stress, and psychological well-being scores as continuous outcome variables. For the depression model, the candidate predictive features were emotional dysregulation, loneliness, social media addiction, academic stress, family support, physical activity, age, and gender. For the psychological well-being model, the candidate predictive features were emotional dysregulation, loneliness, social media addiction, self-esteem, academic stress, family support, physical activity, age, and gender. For the anxiety and perceived stress models, the candidate predictive features were emotional dysregulation, loneliness, social media addiction, self-esteem, sleep quality, academic stress, family support, physical activity, age, and gender.

Multiple linear regression analyses were initially conducted as benchmark models. Subsequently, Random Forest Regression, Support Vector Regression (SVR), and Extreme Gradient Boosting Regression (XGBoost) models were trained and compared using the corresponding outcome-specific feature sets. Hyperparameter optimization was conducted within the training data using cross-validation and the predefined search spaces. The original train–test partition was retained to ensure consistency in the evaluation framework.

The hyperparameter search space and representative optimal configurations are presented in **Supplementary Table 1**. The search space included parameters such as learning rate, maximum depth, and *n*_estimators for XGBoost and Random Forest, and regularization parameters for SVR. Model performance was evaluated on the held-out test set using R², root mean squared error (RMSE), and mean absolute error (MAE). To quantify the uncertainty of model performance, 95% percentile bootstrap confidence intervals (CIs) for R² were calculated using 1,000 resamples.

To improve model interpretability, the TreeSHAP algorithm, specifically optimized for tree-based machine learning, was applied to the best-performing XGBoost model for each outcome using the corresponding outcome-specific feature set. SHAP values were calculated to estimate the contribution of each eligible predictor to model predictions and to identify shared and outcome-specific predictive features. Feature-importance rankings were subsequently compared across the five cross-validation folds to assess their stability across data partitions (37). Statistical significance for conventional analyses was set at *p* <.05.

## 3 Results

A total of 1,244 adolescents were initially invited to participate in the study. Of these, 156 students were excluded 95 declined participation or were absent on the day of assessment, and 61 were excluded due to extensive missing survey data (> 20% missing items). This yielded a final analytic sample of 1,088 participants (response rate: 87.46%). Preliminary attrition analyses revealed no significant differences in mean age (*p* = 0.33) or gender distribution (*p* = 0.57) between the final analytic sample and the excluded students, suggesting minimal non-response bias. Furthermore, preliminary analyses indicated no significant differences in primary mental health outcomes between adolescents in urban and suburban schools (all *p* > 0.05). Within the final analytic sample (N = 1,088), the overall item-level missingness remaining after participant exclusion was low (1.8%) prior to MICE imputation. The sample was relatively balanced in terms of gender, comprising 567 females (52.11%) and 521 males (47.89%). The mean age of the participants was 15.61 ± 1.47 years (**Table 1**).

**Table 1:** Demographic characteristics and descriptive statistics of the sample (N = 1,088).

Variable		n (%)	Mean ± SD
Gender	Male	521 (47.89%)	
	Female	567 (52.11%)	
Age (years)			15.61 ± 1.47
Depression			8.73 ± 5.20
Anxiety			7.92 ± 4.89
Stress			19.37 ± 6.43
Well-being			13.83 ± 4.92
Emotional Dysregulation			42.19 ± 10.34
Loneliness			45.66 ± 11.20
Social Media Addiction			17.94 ± 5.12
Self-esteem			28.40 ± 6.08
Sleep Quality			6.83 ± 3.10
Academic Stress			52.37 ± 9.87
Family Support			21.75 ± 5.01
Physical Activity			41.27 ± 15.45

As presented in **Table 1**, participants reported moderate levels of psychological distress and relatively low psychological well-being. Specifically, the mean scores were 8.73 ± 5.20 for depressive symptoms, 7.92 ± 4.89 for anxiety, and 19.37 ± 6.43 for perceived stress. The average psychological well-being score was 13.83 ± 4.92. Furthermore, the psychosocial predictors exhibited expected variability across the sample. Emotional dysregulation (42.19 ± 10.34) and loneliness (45.66 ± 11.20) demonstrated moderate-to-high variability. Participants also reported notable levels of academic stress (52.37 ± 9.87) and moderate social media addiction (17.94 ± 5.12), alongside mild sleep disturbances (6.83 ± 3.10). Regarding protective factors, the mean scores for self-esteem and perceived family support were 28.40 ± 6.08 and 21.75 ± 5.01, respectively, while physical activity levels were relatively low across the sample (41.27 ± 15.45).

Pearson correlation analyses showed numerous significant associations among the study variables. (**Table 2**). All primary mental health variables were significantly interrelated. Depression demonstrated moderate to strong positive associations with anxiety (*r* = 0.61, *p* < 0.001) and perceived stress (*r* = 0.59, *p* < 0.001), while showing a moderate negative correlation with psychological well-being (*r* = −0.54, *p* < 0.001).

**Table 2:** Pearson correlation matrix of study variables.

Variable	1	2	3	4	5	6	7	8	9	10	11	12
1. Depression	–											
2. Anxiety	0.61***	–										
3. Stress	0.59***	0.60***	–									
4. Well-being	-0.54***	-0.51***	-0.45***	–								
5. Emotional dysregulation	0.56***	0.53***	0.47***	-0.42***	–							
6. Loneliness	0.48***	0.45***	0.44***	-0.49***	0.41***	–						
7. Social media addiction	0.24***	0.26***	0.20***	-0.17***	0.22***	0.29***	–					
8. Self-esteem	-0.45***	-0.39***	-0.37***	0.51***	-0.38***	-0.49***	-0.14***	–				
9. Sleep Quality	0.33***	0.35***	0.32***	-0.25***	0.26***	0.22***	0.29***	-0.19***	–			
10. Academic Stress	0.28***	0.36***	0.49***	-0.22***	0.25***	0.18***	0.15***	-0.23***	0.21***	–		
11. Family Support	-0.29***	-0.22***	-0.19***	0.38***	-0.25***	-0.45***	-0.11***	0.35***	-0.15***	-0.12***	–	
12. Physical Activity	-0.15***	-0.11***	-0.12***	0.19***	-0.06	-0.11***	-0.04	0.15***	-0.11***	-0.03	0.11***	–

N = 1,088; Higher scores in Sleep Quality (PSQI) indicate poorer sleep. *p < 0.05; **p < 0.01; ***p < 0.001.

Among the psychosocial predictors, emotional dysregulation showed the strongest positive correlations with all adverse mental health outcomes, particularly depression (r = 0.56, p < 0.001) and anxiety (*r* = 0.53, *p* < 0.001). Loneliness was also positively associated with depression (*r* = 0.48, *p* < 0.001), anxiety (*r* = 0.45, *p* < 0.001), and stress (*r* = 0.44, *p* < 0.001), while exhibiting a negative correlation with psychological well-being (*r* = −0.49, *p* < 0.001).

Social media addiction showed small-to-moderate positive correlations with depression (*r* = 0.24, *p* < 0.001), anxiety (*r* = 0.26, *p* < 0.001), and stress (*r* = 0.20, *p* < 0.001). Self-esteem was consistently negatively associated with all negative mental health outcomes and positively associated with psychological well-being (*r* = 0.51, *p* < 0.001). Higher PSQI scores, indicating poorer sleep quality, were positively correlated with psychological distress and negatively associated with well-being.

Academic stress demonstrated moderate positive correlations with depression (*r* = 0.28, *p* < 0.001), anxiety (*r* = 0.36, *p* < 0.001), and stress (*r* = 0.49, *p* < 0.001). Family support was negatively associated with distress variables and positively associated with psychological well-being. Physical activity showed weak but significant associations with mental health outcomes.

Prior to evaluating test set performance, sensitivity analyses confirmed that utilizing the MICE-imputed dataset yielded negligible differences in predictive performance compared to the complete-case dataset (N = 932) (average ΔR² < 0.02 across all models), supporting the robustness of the imputation strategy (**Supplementary Table 2**).

**Table 3** presents the performance metrics evaluated on both the training and independent test sets. As expected, Random Forest showed the largest train–test R² gaps across the four outcomes, whereas XGBoost showed smaller gaps and higher test-set performance. Across all four mental health outcomes, XGBoost consistently achieved the highest out-of-sample predictive performance while demonstrating substantially less overfitting than Random Forest models.

**Table 3:** Model performance comparison grouped by outcome.

Outcome	Model	Training R^2^	Test R^2^ (95% CI)	RMSE	MAE
Depression	Linear Regression	0.35	0.33 [0.28, 0.37]	4.35	3.42
	SVR	0.41	0.38 [0.33, 0.42]	4.18	3.25
	Random Forest	0.81	0.44 [0.39, 0.48]	3.95	3.10
	XGBoost	0.63	0.49 [0.44, 0.53]	3.52	2.81
Anxiety	Linear Regression	0.39	0.37 [0.32, 0.41]	3.90	3.01
	SVR	0.45	0.43 [0.38, 0.47]	3.68	2.88
	Random Forest	0.85	0.49 [0.44, 0.53]	3.40	2.69
	XGBoost	0.68	0.55 [0.50, 0.59]	3.04	2.47
Stress	Linear Regression	0.47	0.45 [0.40, 0.49]	4.45	3.48
	SVR	0.54	0.51 [0.46, 0.55]	4.12	3.21
	Random Forest	0.90	0.56 [0.51, 0.60]	3.88	3.01
	XGBoost	0.75	0.60 [0.56, 0.64]	3.71	2.87
Well-being	Linear Regression	0.37	0.35 [0.30, 0.39]	4.05	3.18
	SVR	0.44	0.41 [0.36, 0.45]	3.86	3.02
	Random Forest	0.79	0.46 [0.41, 0.51]	3.55	2.81
	XGBoost	0.61	0.50 [0.45, 0.54]	3.38	2.68

Training R² was calculated on the training dataset after hyperparameter optimization. Test R² values are reported with 95% percentile bootstrap confidence intervals based on 1,000 resamples of the independent test dataset. Outcome-specific feature sets were used to exclude predictors with substantial direct measurement overlap with the respective outcomes.

To elucidate the relative contribution of each predictor, SHAP summary plots were generated for the best-performing XGBoost models (**Figure 1**). Analysis of global feature importance showed that emotional dysregulation and loneliness were consistently among the highest-ranked features across all four mental health outcomes. Beyond these shared predictive features, the models captured distinct outcome-specific patterns. The depression model was primarily driven by emotional dysregulation, loneliness, and academic stress. In contrast, anxiety was most strongly influenced by loneliness, followed by emotional dysregulation and social media addiction.

**Figure 1:**
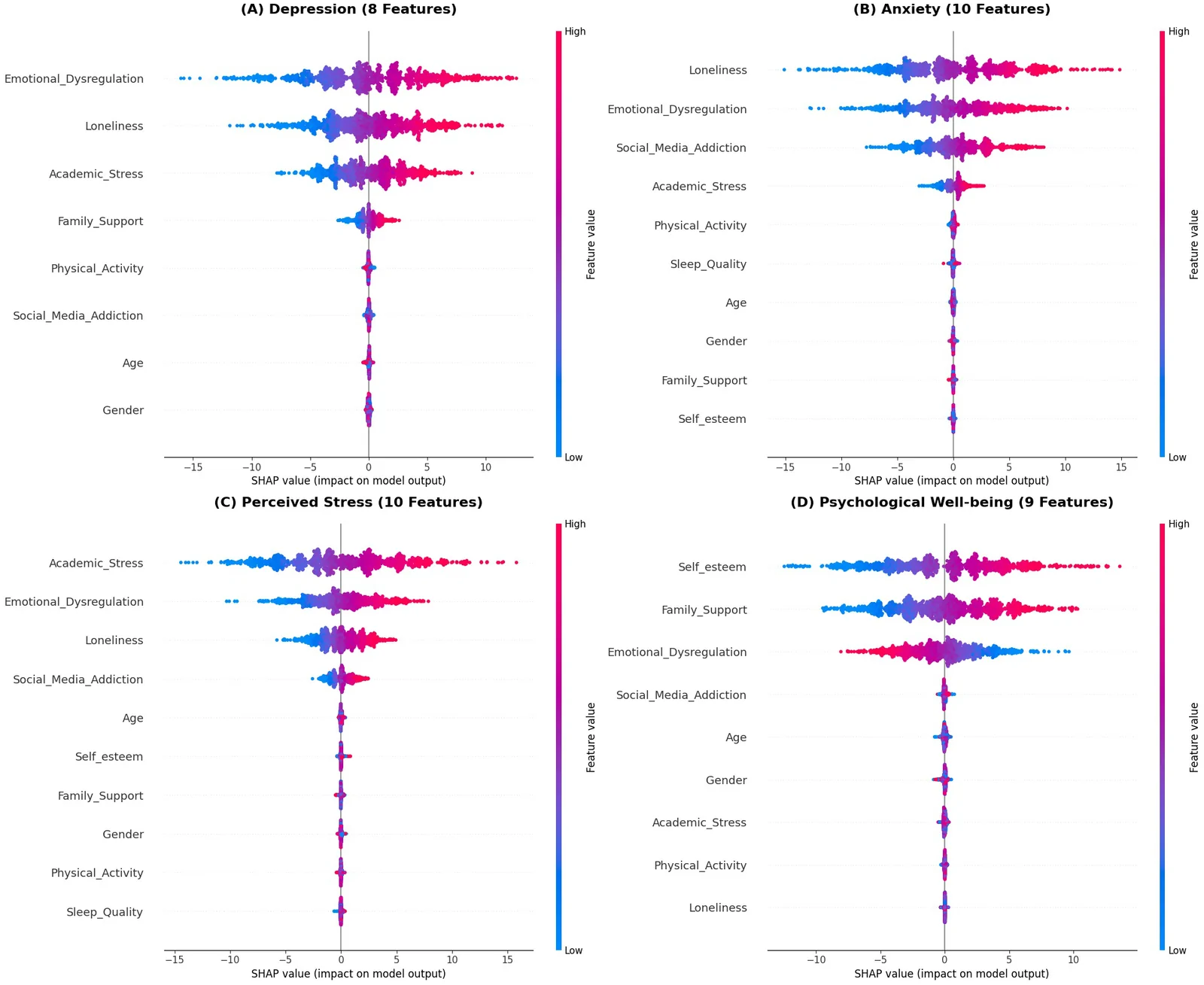
SHAP summary plots illustrating feature importance across the four XGBoost models. Panels depict the contribution of predictors to **(A)** depression, **(B)** anxiety, **(C)** perceived stress, and **(D)** psychological well-being. Variables are ranked according to their mean absolute SHAP values. The horizontal axis represents the impact of each feature on model predictions, whereas color gradients indicate feature values ranging from low (blue) to high (red).

For perceived stress, academic stress emerged as the highest-ranked feature, whereas psychological well-being was primarily associated with high self-esteem, strong family support, and an inverse relationship with emotional dysregulation. To confirm that these SHAP interpretations were not artifacts of a single data split, feature importance rankings were systematically evaluated across all five cross-validation folds. Across the folds, emotional dysregulation and loneliness consistently appeared among the top three SHAP-ranked features, indicating stable feature importance across different training subsets (**Supplementary Table 3**).

Demographic variables, specifically age and gender, exhibited the lowest predictive importance across all four models compared to the psychosocial features. Examination of SHAP dependence plots (**Figure 2**) revealed nonlinear associations between key predictors and mental health outcomes. Loneliness demonstrated a nonlinear association with the predicted outcome, with SHAP values increasing more markedly at moderate-to-high levels. Similarly, social media addiction showed a nonlinear pattern, with larger changes in SHAP values at higher levels of social media use. In contrast, emotional dysregulation demonstrated a relatively linear pattern across its observed range.

**Figure 2:**
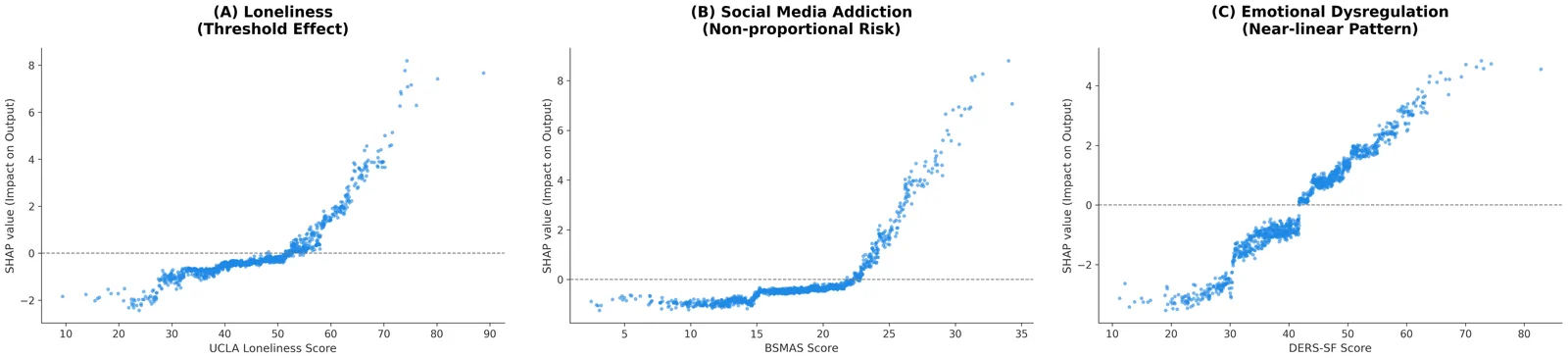
SHAP dependence plots illustrating the relationships between selected psychosocial predictors and model predictions. Panel **(A)** illustrates the nonlinear association between loneliness and model predictions, Panel **(B)** illustrates the nonlinear pattern associated with social media addiction, and Panel **(C)** shows the near-linear association between emotional dysregulation and model output. Positive SHAP values indicate greater contributions to the predicted outcome, whereas negative values indicate reduced contributions.

## 4 Discussion

The present study found that emotional dysregulation and loneliness were the most consistently important predictive features across multiple mental health outcomes, including depression, anxiety, perceived stress, and psychological well-being. Across the XGBoost models, these two factors emerged as shared predictive features across outcomes, while self-esteem, academic stress, and sleep quality showed more outcome-specific patterns of predictive importance. In contrast, demographic characteristics such as age and gender contributed relatively little to model performance, suggesting that psychosocial characteristics were associated with substantially more variation in the study outcomes within this sample. One possible explanation is that both emotional dysregulation and loneliness reflect broad psychosocial processes rather than disorder-specific characteristics. Difficulties regulating emotions may influence how adolescents interpret and respond to everyday challenges (38), whereas loneliness reflects unmet social and emotional needs during a developmental period in which peer relationships become increasingly central (39). Together, these findings suggest that emotional regulation capacity and perceived social connectedness may represent broad dimensions of adolescent psychosocial functioning that cut across multiple mental health domains rather than being uniquely associated with a single outcome. Sleep quality warrants particular methodological consideration in interpreting the present findings. Because the PSQI includes content related to sleep disturbance that directly overlaps with the sleep-related item of the PHQ-9, it was excluded from the depression model to minimize target leakage arising from shared measurement content. Accordingly, the present analysis does not estimate the predictive contribution of sleep quality to depressive symptom scores. The PSQI was retained only in outcome models for which no direct item- or scale-level measurement overlap with the outcome measure was identified.

In contrast, physical activity demonstrated relatively weak predictive importance across all models. Although previous literature has consistently linked physical activity to better mental health outcomes (40), the present findings suggest that the relatively low feature importance observed for physical activity may reflect the greater relative importance of other psychosocial characteristics included in the model. Another possible explanation relates to the distinction between general physical activity and structured sport participation. The measure used in this study primarily captured general physical exertion, which may lack the inherent psychosocial benefits—such as peer support, social connectedness, and structured goal-setting—typically associated with organized sports. Consequently, solitary or unstructured physical activity may not show associations of similar magnitude against psychosocial vulnerabilities like loneliness.

Social media addiction showed small but consistent associations with anxiety and depression, with nonlinear effects observed at higher usage levels. One possible explanation is that occasional or moderate social media use may not be inherently detrimental and may even facilitate peer interaction (41). However, at higher levels of engagement, excessive use may interfere with sleep, face-to-face relationships, and emotional self-regulation, thereby showing stronger statistical associations with poorer mental health outcomes (42). This pattern aligns with recent evidence suggesting that problematic engagement with social media may be better characterized by behavioral dependence and excessive use than by overall exposure alone (43).

From a methodological perspective, the superior performance of XGBoost compared to linear regression and SVR highlights the potential value of nonlinear modeling approaches for psychological datasets in which complex and potentially nonlinear relationships may exist among predictors. Psychological and behavioral characteristics rarely operate independently. Their relationships are often nonlinear and interactive, making ensemble tree-based methods better suited to capture these complex patterns than conventional linear models (44). The integration of SHapley Additive exPlanations (SHAP) further enabled interpretability, allowing clearer interpretation of both shared and outcome-specific feature importance.

Although the present findings should not be interpreted as evidence of causal relationships, they provide useful information regarding the relative importance of psychosocial features associated with adolescent mental health. Explainable machine learning approaches may assist researchers and practitioners in characterizing adolescent mental health profiles marked by elevated emotional dysregulation, loneliness, academic stress, and other psychosocial vulnerabilities. Such information may help guide future hypothesis-driven research and support the development of comprehensive school-based mental health assessment strategies. At the same time, these findings should be viewed primarily as hypothesis-generating rather than clinically definitive because the observed associations require confirmation in longitudinal and externally validated studies.

Although XGBoost achieved the highest predictive performance across all four outcomes (test-set R² = 0.49–0.60), a substantial proportion of outcome variance remained unexplained. This finding highlights the multifactorial nature of adolescent mental health and suggests that additional biological, developmental, family, school, and environmental influences, which were not included in the present models, are also likely to contribute to these outcomes. Consequently, these models should be considered complementary tools for research and population-level mental health assessment rather than replacements for comprehensive clinical assessment. This finding also illustrates the complexity of adolescent mental health, indicating that no single psychosocial profile is likely to account for psychological functioning in isolation.

Several limitations should be acknowledged. First, the cross-sectional design precludes causal inferences regarding the observed associations. Second, the reliance on self-reported measures may introduce common method bias and social desirability effects. Third, the internal consistency of some measures, particularly the family support and physical activity scales, was slightly below the conventional threshold, which may have attenuated the observed associations for these variables. Fourth, although the sample was relatively large and diverse within a specific metropolitan area, generalizing these findings to adolescents in different cultural or educational contexts should be done with caution. Furthermore, family socioeconomic characteristics (e.g., parental education, household income, and parental occupation) were not collected. Because socioeconomic conditions are known to be associated with adolescent mental health, future studies should examine whether these factors improve model performance and generalizability. Although schools were randomly selected from urban and suburban districts and represented different academic achievement levels, school-level contextual characteristics (e.g., educational climate, institutional resources, or school-specific academic pressure) were not assessed. In addition, adolescents from rural areas were not represented; therefore, caution is warranted when generalizing these findings beyond similar metropolitan settings. Because the PSQI was excluded from the depression model to avoid direct measurement overlap with the PHQ-9, the present analysis does not estimate the predictive contribution of sleep quality to depressive symptoms. Future studies using measurement designs that minimize such content overlap may further clarify the relationship between sleep characteristics and depression. Finally, all models were developed and evaluated within a single metropolitan sample. Although internal validation was performed, additional external validation in independent populations is needed before the findings can be generalized more broadly. Future longitudinal and multi-site studies are warranted to validate the observed predictive structures and further unpack the temporal dynamics of these psychosocial features.

## 5 Conclusion

In summary, explainable machine learning models consistently highlighted emotional dysregulation and loneliness as the psychosocial features with the greatest relative importance across multiple adolescent mental health outcomes, while sleep quality, academic stress, and self-esteem showed more outcome-specific patterns of predictive importance. These findings highlight the value of integrating explainable machine learning techniques into adolescent mental health research to characterize complex psychosocial patterns while maintaining model transparency. Because the present study was cross-sectional, the observed relationships should be interpreted as associative rather than causal. Future longitudinal and externally validated studies are needed to determine the temporal stability and generalizability of these findings.

## Data Availability

The raw data supporting the conclusions of this article will be made available by the authors, without undue reservation.
