# Supplementary material for: Predicting multiple mental health outcomes in adolescents using explainable machine learning models
Source: Front Psychiatry. 2026 Sep 18;17:1923390. doi: 10.3389/fpsyt.2026.1923390 (PMC13631644; doi:10.3389/fpsyt.2026.1923390)
Supplement: Supplementary file 1 [file Table1.docx]

**Supplementary Table S1: Hyperparameter Search Space and Representative Optimal Configuration (Depression Model)**

| Algorithm | Hyperparameter | Search Space (GridSearchCV) | Optimal Configuration (Depression) |
| --- | --- | --- | --- |
| XGBoost | n_estimators | [100, 200, 300, 400, 500] | 300 |
|  | max_depth | [3, 5, 7, 10] | 7 |
|  | learning_rate | [0.01, 0.05, 0.1, 0.2] | 0.05 |
|  | subsample | [0.6, 0.8, 1.0] | 1.0 |
|  | colsample_bytree | [0.6, 0.8, 1.0] | 1.0 |
| Random Forest | n_estimators | [100, 200, 300, 500] | 500 |
|  | max_depth | [None, 5, 10, 15, 20] | 15 |
|  | min_samples_split | [2, 5, 10] | 2 |
|  | min_samples_leaf | [1, 2, 4] | 1 |
| SVR | C | [0.1, 1, 10, 100] | 100 |
|  | gamma | ['scale', 'auto', 0.01, 0.1] | 'scale' |
|  | kernel | ['linear', 'rbf'] | 'rbf' |

*Note. The optimal configurations shown are those selected for the depression model; configurations for the other outcomes were selected independently using the same predefined search spaces. Optimal parameters for anxiety, stress, and well-being models varied slightly but remained within the defined search space.*
