# Supplementary material for: Predicting multiple mental health outcomes in adolescents using explainable machine learning models
Source: Front Psychiatry. 2026 Sep 18;17:1923390. doi: 10.3389/fpsyt.2026.1923390 (PMC13631644; doi:10.3389/fpsyt.2026.1923390)
Supplement: Supplementary file 2 [file Table2.docx]

**Supplementary Table S2: Sensitivity Analysis Comparing Predictive Performance on MICE-Imputed versus Complete-Case Datasets**

| Outcome | Algorithm | Complete-Case R^2^ (N = 932) | MICE-Imputed R^2^ (N = 1,088) | ΔR^2^ |
| --- | --- | --- | --- | --- |
| Depression | Linear Regression | 0.31 | 0.33 | +0.02 |
|  | SVR | 0.37 | 0.38 | +0.01 |
|  | Random Forest | 0.43 | 0.44 | +0.01 |
|  | XGBoost | 0.48 | 0.49 | +0.01 |
| Anxiety | Linear Regression | 0.36 | 0.37 | +0.01 |
|  | SVR | 0.41 | 0.43 | +0.02 |
|  | Random Forest | 0.48 | 0.49 | +0.01 |
|  | XGBoost | 0.54 | 0.55 | +0.01 |
| Stress | Linear Regression | 0.44 | 0.45 | +0.01 |
|  | SVR | 0.49 | 0.51 | +0.02 |
|  | Random Forest | 0.55 | 0.56 | +0.01 |
|  | XGBoost | 0.59 | 0.60 | +0.01 |
| Well-being | Linear Regression | 0.34 | 0.35 | +0.01 |
|  | SVR | 0.39 | 0.41 | +0.02 |
|  | Random Forest | 0.45 | 0.46 | +0.01 |
|  | XGBoost | 0.49 | 0.50 | +0.01 |

*Note. Complete-case models were trained and evaluated using participants with complete data for all variables included in the corresponding outcome-specific model (N = 932). MICE-imputed models included all eligible participants (N = 1,088) and correspond to the final models reported in the main manuscript. ΔR² represents the difference in test-set R² between the MICE-imputed and complete-case analyses. The small differences in R² indicate that predictive performance was robust to the choice of missing-data handling approach.*
