# Supplementary material for: Predicting multiple mental health outcomes in adolescents using explainable machine learning models
Source: Front Psychiatry. 2026 Sep 18;17:1923390. doi: 10.3389/fpsyt.2026.1923390 (PMC13631644; doi:10.3389/fpsyt.2026.1923390)
Supplement: Supplementary file 3 [file Table3.docx]

**Supplementary Table S3: Stability of SHAP Feature Rankings Across 5-Fold Cross-Validation (XGBoost Model for Depression)**

| Feature | Fold 1 Rank | Fold 2 Rank | Fold 3 Rank | Fold 4 Rank | Fold 5 Rank | Mean Rank ± SD |
| --- | --- | --- | --- | --- | --- | --- |
| Emotional Dysregulation | 1 | 1 | 2 | 1 | 1 | 1.20 ± 0.45 |
| Loneliness | 2 | 2 | 1 | 2 | 2 | 1.80 ± 0.45 |
| Academic Stress | 3 | 4 | 3 | 3 | 4 | 3.40 ± 0.55 |
| Family Support | 4 | 3 | 4 | 5 | 3 | 3.80 ± 0.84 |
| Social Media Addiction | 5 | 5 | 5 | 4 | 5 | 4.80 ± 0.45 |

*Note. The table presents the rank order of the top five predictive features based on mean absolute SHAP values across each fold of the 5-fold cross-validation process for the depression model (excluding PSQI and RSES). The relatively small standard deviations of the mean rankings support the stability of feature importance across the cross-validation folds.*
